# Ketamine and Pulmonary Oedema–Report of Two Cases

**Published:** 2009-08

**Authors:** S Parthasarathy, M Ravishankar, S Selvarajan, T Anbalagan

**Affiliations:** 1 Chief Anaesthesiology, Govt. Dist. Hq Hospital, Kumbakonam; 2 Professor, Mahatma Gandhi Medical College and Research Institute, Pondicherry; 3 Consultant Surgeon; 4 Consultant Anaesthesiologists

**Keywords:** Pulmonary oedema, Perioperative, Ketamine

## Abstract

**Summary:**

Perioperative pulmonary oedema is one of the most challenging complications faced by anaesthesiologists. In most of the instances, coronary artery disease, valvular heart diseases, hypertension may precipitate pulmonary oedema due to increased hydrostatic pressure while acid aspiration, airway obstruction may cause it due to increased vascular permeability. In a few instances, acute pulmonary oedema can present in an otherwise healthy patient to cause diagnostic difficulties. We report two such cases of intra operative pulmonary oedema with the use of ketamine which were identified and managed successfully. The most probable cause is also described.

## Introduction

Pulmonary oedema is the abnormal accumulation of fluid in the interstitial or alveolarspaces of the lung. It occurs for a number of reasons which can be explained on the basis of a disturbance in the normal Starling equation. It involves changes in hydrostatic or oncotic pressure across the alveolar membrane or in the permeability of the alveolar membrane such that fluid moves across from the capillaries into the alveolar space^1^. Traditional teaching is that pulmonary oedema occurring in patients tends to be cardiac in aetiology, with usually left heart dysfunction causing back pressure across the pulmonary system, resulting in extravasation of fluid into the alveoli. In patients undergoing anaesthesia, increased afterload as in neurogenic pulmonary oedema and othernon cardiogenic causes are also encountered^2^‐^4^. Ketamine is a widely used anaesthetic agent with proven circulatory changes. We encountered two cases of intraoperative pulmonary oedema with the use of ketamine as the anaesthetic agent which are reported below.

## Case Reports

**Case 1.** A 27-year-old 60 kg healthy male presented for testicular biopsy. Preoperative investigations including a routine ECG were normal. Atropine 0.5 mg, diazepam 5 mg and ketamine 100 mg were administered intravenously. Noninvasive blood pressure, pulse oximeter and ECG monitoring were applied. He was given 100% O_2_ with a normalregular spontaneous respiration. Pulse rate was 85/min. and blood pressure was maintained at 130/80 mm Hg. Ten minutes later the patient desaturated and SpO_2_ came to around 65-70%. Respiration was shallow with a rate of around 35/min. There was neither suprasternal nor supraclavicular retraction. There was no rocking horse movement of the chest to prove respiratory obstruction. There was neither active wheeze nor vomitus in the mouth to suggest acid aspiration. The heart rate increased to 130/min. with scattered basal crepitations. The blood pressure was 120/80. As the patient desaturated further, he was intubated immediately. The patient allowed intubation without muscle relaxants and given positive pressure ventilation (IPPV). In a short time, the bag was tight with increased resistance to ventilation and soon there was pink frothy fluid from the endo tracheal tube. He was managed with intravenous frusemide 100 mgand IPPV with pressure controlled ventilation continued. With this, the clinical profile improved. The heart rate settled down between 90–100/min. The conscious status progressed and in the next hour, IPPV was discontinued and the patient was maintained with 'T' piece with satisfactory respiratory rate of around 20-25/min and stable haemodynamics. The patient was extubated after three hours. Postoperative chest x ray was normal. Our town doesn't have the facility of blood gas analysis. He was discharged in two days in normal condition.

**Case 2.** A two-year-old healthy male child weighing 12 kg presented for circumcision. Routine preoperative investigations were normal. Ketamine 25 mg, atropine 0.2 mg and diazepam 2 mg were administered intravenously. Pulse oximeter and precordial stethoscope were the monitors applied. For the first ten minutes the baby was normal with SpO_2_ of 100% and heart rate of around 100/min. The patient started to desaturate slowly in the next 10 minutes with SpO_2_ decreasing to 50%. There was neither clinical airway obstruction nor aspiration. Respiratory rate went up to 45/min with minimal basal crepitations. The clinical scenario worsened to allow intubation. Endotracheal intubation was done and IPPV instituted with a tight bag and frothy pink fluid was coming from the tube. Frusemide 10mg with dexamethasone 1 mg was given intravenously. IPPV was continued in the PCV mode according to our accepted protocols and settings for the next two hour. With this management, the patient improved significantly with regard to respiratory rate, SpO_2_ and added sounds in the lung. The conscious status came to normal. Later the child was extubated after a T piece trial of one hour. The patient had a totally uneventful postoperative period. A probable diagnos is of ketamine induced pulmonary oedema was made.

## Discussion

Ketamine is an induction agent with distinct analgesia, rapid onset of action with short duration. It is used extensively in developing countries because of its low cost. Ketamine is a potent sympathomimetic agent. In normal patients, it produces increased pulmonary artery pressure, increased pulmonary vascular resistance, and increased right ventricular stroke work. Ketamine causes tachycardia, hypertension and bronchodilation. These effects mimic sympathetic stimulation^5^. Ketamine increases heart rate, cardiac output and pulmonary arterialpres sure (PAP). The rise in PAP is more consistent than the rise in PVR; resistance changes were greatest in patients with elevated resting PVR^6^. Stimulation of CNS (central nervous system) to increase sympathetic nervous system outflow is one of the proposed mechanism. The other is the inhibition of norepinephrine uptake in the postganglionic sympathetic nerve endings to elevate plasma catecho lamine levels. These may explain the onset of pulmonary edema in cocaine addicts with ketamine^7^. We neither encountered active wheezing nor aspirate in the mouth or orophatynx. Hence in our cases the possibility of pulmonary oedema either due to respiratory obstruction^8^ or aspiration was ruled out on clinical grounds. As there is a distinct analgesia with the use of ketamine and the surgery being minor we did not use any added analgesics. Hence the diagnosis was centered on two possible causes of pulmonary oedema.

1. Ketamine itself producing pulmonary oedema either by direct stimulation of CNS to effect a sympathetic discharge or inhibition of norepinephrine uptake in the postganglionic sympathetic nerve endings to elevate plasma catecholamine levels. At least one such case is reported in a patient with coronary artery disease^9^ and another in a healthy child for dressing of burns wound^10^. Hypertension and pulmonary oedema are more likely after ketamine in patients with substance abuse^11^ and use of ketamine should be cautious and in such patients.
2. Propylene glycol^12^, an organic solvent in the preparation of diazepam is reported to cause stimulation of vasoactive substances to result in pulmonary oedema. Reports of this solvent producing such complication are reported in animals. Because of its extreme rarity and lack of human reports with propylene glycolwe concluded the probable cause could be due to the use of ketamine.

Ketamine, an anaesthetic agent with unique advantages has got widespread use in clinical practice. We report two cases of pulmonary oedema following ketamine use to caution the casual users of the drug and to create awareness about this possibility in undiagnosed cases of perioperative pulmonary oedema.
